# Pilot trial of The Living Well Toolkit: qualitative analysis and implications for refinement and future implementation

**DOI:** 10.1186/s12913-020-4920-5

**Published:** 2020-01-30

**Authors:** Suzie Mudge, Ann Sezier, Deborah Payne, Greta Smith, Nicola Kayes

**Affiliations:** Centre for Person Centred Research, Auckland University of Technology, Auckland, New Zealand

**Keywords:** Implementation, Long-term conditions, Normalisation process theory, Person-centred, Toolkit

## Abstract

**Background:**

Following a neurological event, people’s long-term health and well-being is hampered by a system that struggles to deliver person-centred communication and coordinated care and fails to harness individual and family capability to live well with the condition. We aimed to implement and evaluate a toolkit package to support these processes for people with long-term neurological conditions.

**Methods:**

This is a multi-phased study drawing on the principles of participatory research. In this pilot phase, the toolkit package was introduced to clinicians, who introduced it to clients in four neurorehabilitation settings (inpatient and community-based). Individual and focus group interviews were carried out with clients (*n* = 10) and clinicians (*n* = 9). Data were categorised by the four components of Normalisation Process Theory (NPT), and data within each component was then coded inductively. This analysis was used to inform revisions to the toolkit package and wider implementation processes.

**Results:**

There was widespread support for the principles underpinning the toolkit package from clients and clinicians. However, it was less clear how the client toolkit could support these principles in clinical practice which impacted buy-in. The flexibility of use of the client toolkit, which we encouraged, made it difficult for clinicians and clients to be clear about its purpose and for clinicians to operationalise in practice. Clinicians and clients identified a number of barriers that limited the time, energy and work users were able or prepared to invest, to the extent that uptake of the toolkit package was modest. Use of the toolkit package appeared more likely when clinicians perceived it to augment existing processes (e.g. goal setting) rather than detract from ‘doing’ therapy. This analysis was used to inform revisions to the toolkit package, including simplification of the client toolkit, development of videos with examples of use and a modular and reflective training package for clinical services. The refinements were intended to improve sense-making and minimise the cognitive barriers associated with implementation of a new intervention.

**Conclusion:**

Understanding how supporting the client toolkit could add value to the therapeutic encounter was necessary for clinicians to invest time and perceive the worth of the toolkit package.

**Trial registration:**

ANZCTR: ACTRN12614000537651. Registered 21 May, 2014.

## Background

Long-term neurological conditions result in significant personal, family and societal burden. Despite advances in acute medical care, research highlights that people’s long term recovery and adaptation is frequently hampered by a system that struggles to deliver good quality care [1–3]. Clients’ expectations of care, including person-centred communication [4], harnessing their own strengths and resources [5] and coordination between services [6–8], are often not translated into routine practice [2, 9]. A change in how we work with people is clearly needed and studies have highlighted that relatively simple changes in how we listen to and work with patients can improve outcome, quality of care and assist people to take charge of their condition resulting in improved health [10–13].

The aim of this project was to operationalise these three processes (person-centred communication, harnessing of consumer’s strengths and resources and coordination of services across the lifespan) throughout the healthcare pathway through the development of a practical and workable toolkit. In Phase 1, we used qualitative descriptive methods to explore the experiences and perspectives of clients, their family, health services and clinicians regarding what was working well and what could be better with respect to these core processes. We constructed five themes from the data that were common to rethinking the three processes. The design and content of the Living Well Toolkit package was based on these themes. A detailed account of phase 1 and the toolkit development has been previously published [14].

**Fig. 1:**
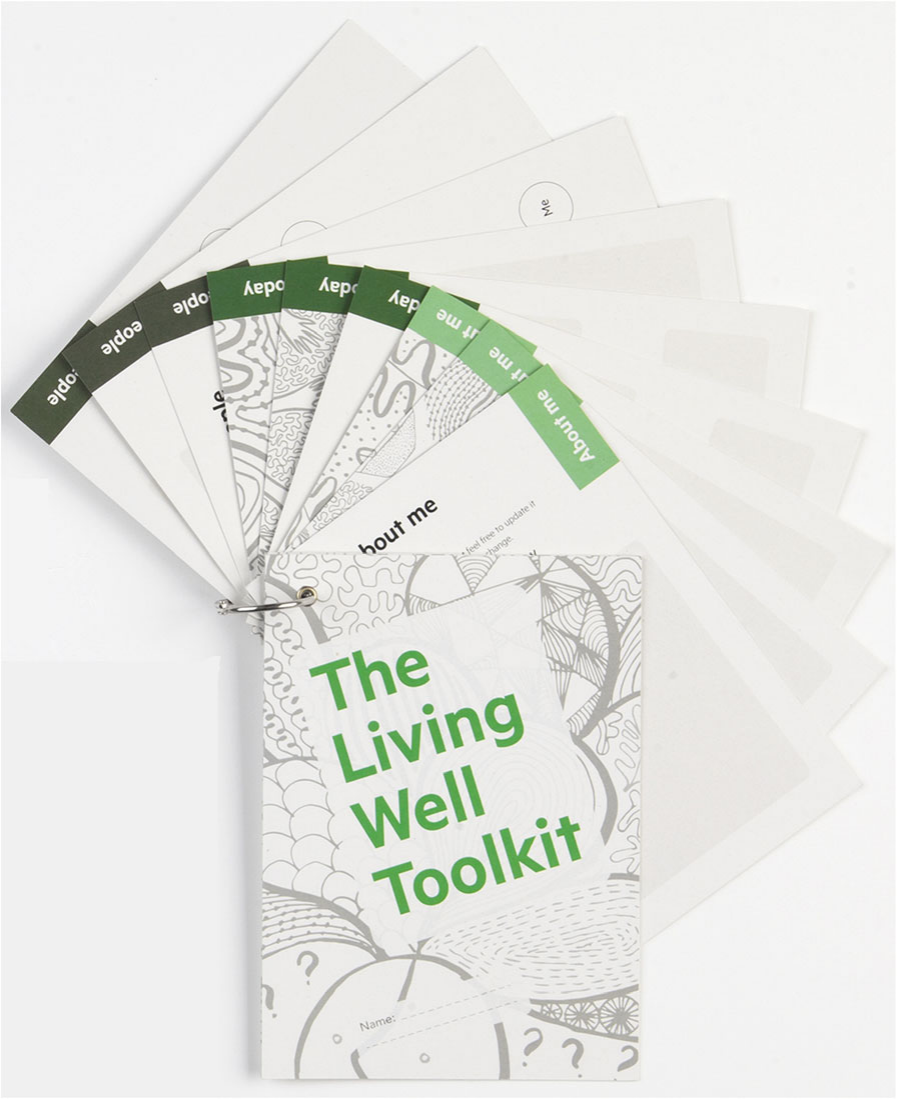
Client toolkit (early version). Developed by our team for this study

**Fig. 2:**
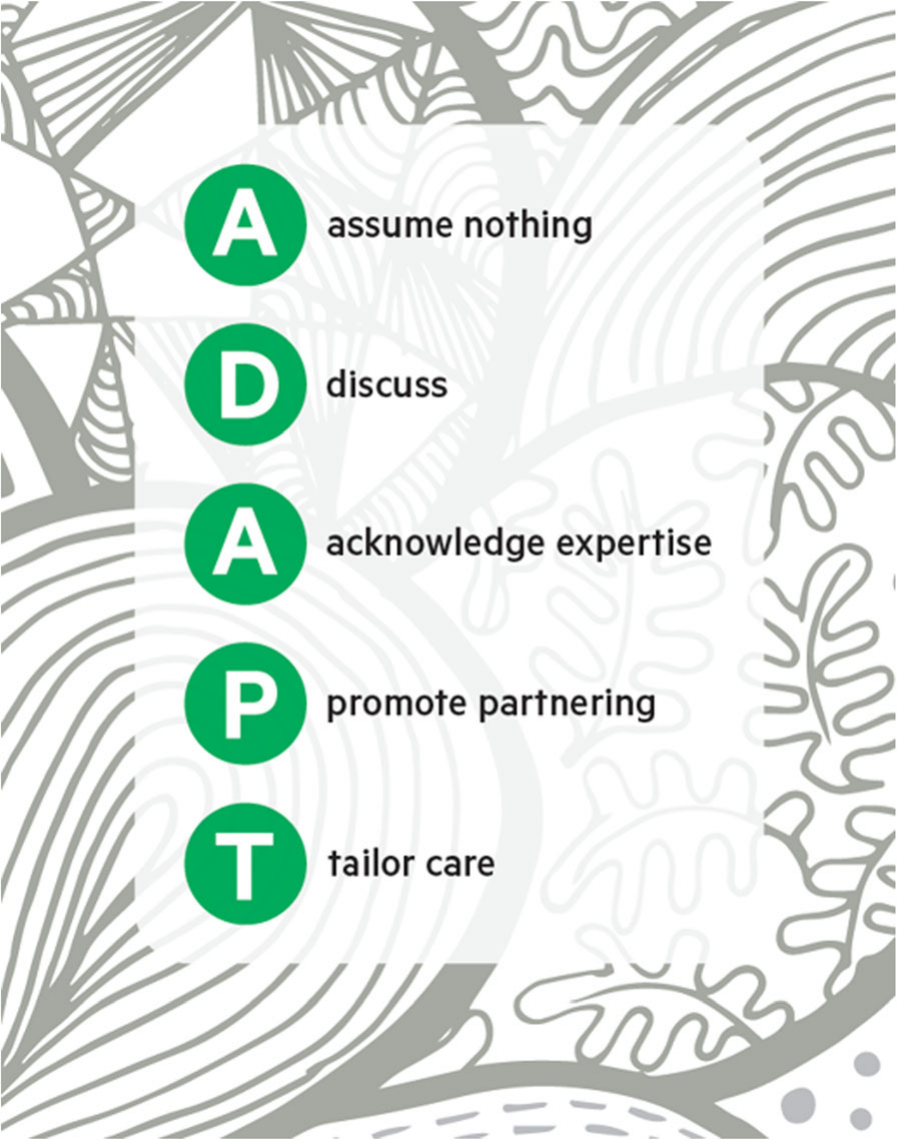
ADAPT prompt card. Developed by our team for this study

The Living Well Toolkit package designed in phase 1 (henceforth called the toolkit package) comprises two components. The first is a paper based toolkit (Fig. 1) with three sections ‘About me’, ‘My needs today’ and ‘People’, designed to be held by the client and used in healthcare interactions as desired (henceforth called ‘client toolkit’). It was designed deliberately to be flexible so that clients can use whichever section(s), whenever and in whatever situation(s) best suited their needs. The second component is a clinicians’ resource that provides a structural support for clinicians to underpin a way of working that is consistent with the client toolkit and the three core processes. We constructed an acronym from the five key principles underpinned by our findings from phase 1 (**A**ssume nothing, **D**iscuss, **A**cknowledge expertise, **P**romote partnering, **T**ailor Care) – ADAPT (Fig. 2) to guide practice, which was incorporated into a prompt card with a central orienting question ‘Who is this person and what do they need from me today?’ We additionally developed an electronic layered clinicians’ resource to provide more detail and examples of the ADAPT acronym in practice as well as quotes from the original data [14]. In this paper, we report on the findings of the pilot study, which was phase 2 of this multi-phased implementation project.

## Methods

### Aim

The aim of phase 2 was to pilot the Living Well Toolkit package in a diversity of settings to explore the personal and clinical utility, and acceptability of the toolkit package and related implementation processes. Our overarching goal was to refine aspects relevant to intervention and implementation to increase the likelihood of future uptake.

### Design

We took a participatory approach underpinned by the Knowledge-­to-­Action framework [15] for the project in its entirety. For this specific phase, we used an acceptability-implementation hybrid design drawing on qualitative methods [16], given the dual focus on utility and acceptability of the toolkit package and related implementation processes. Our design is similar to the hybrid trial type 2 proposed by Curran et al. [16] but acknowledges the pilot stage of the current study. We particularly focused on adapting the intervention to the local context, identifying and promoting any facilitators to use and assessing and responding to barriers to use, consistent with a knowledge translation approach [15]. To this end, we intentionally involved key stakeholders (clients, families and clinicians) to ensure the intervention was workable for all [17, 18].

### Setting

We recruited participants from four facilities in Auckland. Two facilities were rehabilitation wards within large public hospitals. A third facility was a residential rehabilitation facility and the fourth, an outpatient community rehabilitation clinic. These facilities were purposefully sampled for diversity so the toolkit package could be piloted across settings and contexts and at different stages post injury or diagnosis.

### Participants

We anticipated a sample of between 6 and 9 clinicians and 6 and 9 clients would enable an in-depth exploration of a diversity of perspectives sufficient to inform next steps. However, we continued sampling until sufficient perspectives were gathered, drawing on the concept of information power [19]. Clinicians were eligible to participate if they were involved in using the toolkit package as part of this pilot trial in one of the four participating facilities and consented to share their perspectives and experiences of that process. Participating clinicians introduced the client toolkit to clients with a long-term neurological condition over the age of 18 and invited them to participate. Consenting clients were invited to nominate one or more significant person(s) within the client’s circle, who might also be willing to share their views on the utility and acceptability of the client toolkit.

### Procedures

A member of the research team introduced the tookit package to a senior clinician within each healthcare service, who agreed to act as implementation champion for the service. We offered a training session of no longer than an hour to introduce the project and the toolkit package, including purpose and intended use. The limited time allocated to introducing the toolkit package was deliberate with consideration of the need for a process with limited burden and impact on staff and resources for future uptake. The implementation champion and researcher negotiated the preferred mechanism for that locality to introduce the toolkit package to the clinical team (e.g. via a group training session led by the research team or introduction of the toolkit package via the implementation champion). Clinicians willing to implement the toolkit package in their practice and take part in the research were invited to indicate their interest to the research team at the training session or through their implementation champion.

Participating clinicians approached eligible clients to gauge interest in using the client toolkit and participating in the research project. A member of the research team then met with the client to answer questions and to confirm their desire to enter into the study. The timing of introduction of the client toolkit was left to the discretion of the treating clinician. In the acute hospital setting, we suggested that prior to discharge or another transition stage was the ideal timing for introduction of the client toolkit. We also recommended clinicians invite consecutive eligible clients to allow exploration of the utility of the client toolkit for a diversity of clients, rather than only those the clinician perceived might be responsive. In the absence of evidence to inform decisions regarding who might benefit from using the client toolkit, more purposeful selection by clinicians would be a subjective decision and based on assumptions about who is likely to benefit.

### Intervention

The development, structure and intended use of the toolkit package are published elsewhere [14] and briefly described in the introduction. In our interactions with implementation champions and clinical staff, we emphasised the flexible nature of the toolkit package to suit the needs and preferences of clients. Because of this flexibility, we anticipated that the toolkit package could be introduced to the majority of clients.

### Data collection

We collected basic demographic data (gender, ethnicity and rehabilitation setting) for all participants. We also recorded details of primary diagnosis for clients and disciplinary background for clinicians. In semi-structured interviews with the client and nominated significant others, we collected data concerning the utility of the client toolkit. In particular, participants were prompted to share their initial thoughts about the toolkit, their experience of using it (if they had), and feedback about the content, design and format. Interviews occurred within the first three weeks after commencing use of the client toolkit and were repeated three months later. In addition, we invited participating clinicians to take part in an individual or focus group interview to explore their perspectives of implementing and interacting with the client toolkit and the clinicians’ resource.

AS (MPhil) conducted all client and family interviews and NK, DP and SM (each have a PhD) conducted clinician interviews and/or focus groups. All researchers were female and all have considerable qualitative interviewing experience. All client and family participants were unknown to the interviewer, however because the clinician participants were known to some of research team, where possible, an interviewer who was unknown to participants was selected (to allow more freedom to express views) and held at a location of the participants’ choosing. All interviews (between 20 and 60 min) were recorded and transcribed. Interview guides for clients and clinicians are shown in Additional file 1 and Additional file 2 respectively. Participants were not offered their transcript to review given that we argue data is constructed in time and place between researcher and participant.

### Analysis

We tabulated demographic data to illustrate context of participants. We used normalisation process theory (NPT) [20] as the framework to identify key areas for intervention refinement. We selected NPT as it provides a sociological set of tools to understand the three core processes of implementing, embedding and integrating new healthcare practices [20] by identifying promoting and inhibiting factors to illuminate the way in which the intervention becomes ‘normalised’ in practice [21]. Coherence (meaning and sense making by participants), collective action (the work participants do to make the intervention function), cognitive participation (engagement and commitment of participants) and reflexive monitoring (how participants appraise the intervention) are the key components of NPT considered to be the conditions under which new practices become routinely incorporated into practice – or ‘normalised’. Murray et al. [21] developed a set of questions within each of the four components to understand how and why a low back pain intervention was incorporated (or not) into practice. We adapted these questions (Table 1) and they served as analytical questions to identify aspects relevant to intervention and implementation that may impact future uptake. Initially one researcher categorised data by each of the broad NPT components and then inductively coded data within each component using the analytical questions to support interpretation of data. We drew on the general tenets of a directed content analysis approach [22] where an existing theoretical framework informed the initial coding schema. Categorisation and coding were discussed regularly with other team members. New codes were generated for data that did not have an obvious fit with the existing NPT components.

**Table 1:** Questions arising from components of Normalisation Process Theory used to inform data analysis

Component	Questions
Coherence (meaning and sense making by participants)	Is the toolkit package easy to describe?
	Is it clearly distinct from other interventions?
	Does the toolkit package have a clear purpose for all relevant participants?
	Do clinicians and patients have a shared sense of its purpose?
	What are the perceived benefits of the toolkit package and for whom?
	Are these benefits valued by clinicians and patients?
	Does the toolkit package fit with the overall goals and activity of the service?
Cognitive participation (commitment & engagement by participants)	Are target user groups likely to think the toolkit package is a good idea?
	Do clinicians and patients see the point of the toolkit package easily?
	Will clinicians and patients be prepared to invest time, energy and work to use the toolkit package?
Collective action (work participants do to make the intervention function)	How does the toolkit package affect the work of clinicians?
	Does it promote or impede their work?
	Do clinicians require extensive training before they can use the toolkit package?
	How compatible is it with existing work practices?
	What impact will it have on division of labour, resources, power and responsibility between different professional groups?
	Will it fit with the overall goals and activity of the service?
Reflexive Monitoring (participants reflect on or appraise the intervention)	How are clinicians likely to perceive the toolkit package once it has been in use for a while?
	Is it likely to be perceived as advantageous for patients or staff?
	Will it be clear what effects the toolkit package has had?
	Can clinicians or patients contribute feedback about the toolkit package once it is already in use?
	Can the toolkit package be adapted/ improved on the basis of experience?

## Results

In total, 14 clinicians and 14 people with neurological conditions were exposed to the toolkit package. Of these, 9 clinicians and 10 clients agreed to participate and were interviewed about their experiences of the toolkit package, either in a focus group (5 clinicians) or individual interview (10 clients and 4 clinicians). No significant others were nominated by clients. Of the 10 clients that did take part, only two participated in a second follow-up interview at 3 months (*n* = 6 refused, *n* = 2 not contactable). Table 2 provides demographic information related to gender, ethnicity and current rehabilitation setting for all participants. Details of primary diagnosis (for clients) and disciplinary background (for clinicians) are also provided.

**Table 2:** Participant Demographics

Clinicians (*n* = 9)		Clients (*n* = 10)	
Gender	Female: 7 Male: 2	Gender	Female: 3 Male: 7
Ethnicity^a^	NZ European: 5 Māori: 0 Other European: 4	Ethnicity^a^	NZ European: 8 Māori: 3 Other European: 0
Current setting	Hospital: 2 Inpatient rehabilitation: 5 Community rehabilitation: 2	Current setting	Hospital: 3 Inpatient rehabilitation: 5 Community rehabilitation: 2
Discipline	Physiotherapist: 4 Speech language therapist: 3 Occupational therapist: 1 Clinical psychologist: 1	Primary diagnosis	Traumatic brain injury: 5 Stroke: 2 Spinal cord injury: 2 Guillain Barré Syndrome: 1

^a^ Numbers may not add to total as participants were able to identify with more than one ethnicity or elected not to provide these details

### Living Well Toolkit intervention refinement & implementation

The findings are structured according to the four NPT components as described above.

### Coherence

Coherence describes the meaning that users collectively invest in an intervention. In this case, coherence specifically addressed whether the toolkit package made sense to clinicians and clients and their families, whether the purpose of the toolkit package was clear, easy to describe and distinct from other interventions.

Although the client toolkit initially appeared intuitive to clinicians, they struggled to introduce and explain it to clients.

*“… yeah this makes good sense,” and then later on I was thinking, “Right, I will introduce it to a couple of my new clients.” and then thought, “OK, how am I going to do this? And how am I going to explain what it is and describe it?” And that took me quite a while to get my head around*. (Clinician)

Some clients were unclear of the purpose of the client toolkit. One individual thought it was a diary, another thought it was a satisfaction survey and some used it to support specific processes (e.g. for goal setting related to discharge planning). While the client toolkit was designed to be flexibly used, this appeared to create a lack of clarity for both clients and clinicians.

*Well it was explained that it wasn’t a hard and fast thing; I had to definitely figure out things. I could decide even not to do it and then come and say, “I just didn’t feel that I could maybe do it.”* (Client)

Most clients broadly perceived value in the client toolkit, if not for themselves, then for other clients. Some clients felt their communication with clinicians was already excellent and so the client toolkit was not necessary in their specific situation. However most clients agreed that the questions in the client toolkit were congruent with their thinking and were useful as a prompt. Clinicians thought there was value for clients who used the client toolkit in not needing to repeat information. They also believed the toolkit package resonated with a person-centred approach and, in particular, had potential to enhance goal setting processes and a self-management approach.

*It’s actually helping us writing out goals for that person as well because we know what’s important to them and we can include that within their goals which we should be doing anyway. But you know we don’t always…* (Clinician)

Although, on a cognitive level, clinicians understood the toolkit package was intended to improve two-way interaction, in practice, some clinicians focussed on the client toolkit, seeing it as a tool to benefit clients only. Some clinicians and clients also questioned whether it would be *awkward* or *would feel rude* to take the client toolkit to a health provider unaware of the project. Not all clinicians perceived the toolkit package would add value to their practice because they perceived they were already person-centred in their practice.

In summary, both clients and clinicians agreed with the underpinning philosophy of the toolkit package and certain aspects were valued. On the other hand, there was some confusion about the purpose and intended use of the client toolkit and clinicians felt they were not confident to introduce, explain and incorporate it into practice. While we intended for the toolkit to be used flexibly (tailored to meet the unique and specific needs of the individual), this appeared to result in ambiguity for clinicians and clients, potentially contributing to the value proposition of the toolkit being somewhat nebulous.

### Cognitive participation

Cognitive participation includes the extent to which clinicians and clients are prepared to invest time, energy and work into using the toolkit package and whether they think it is a good idea. Various factors were identified as important that either helped or hindered uptake of the toolkit package by clinicians and clients alike. In particular, clinicians weighed up whether the toolkit package resonated with their philosophy of practice. Similarly, clients assessed the client toolkit’s compatibility with their thinking. This congruence with thinking was a critical first assessment for both groups before committing further.

Conversely, both groups also identified barriers that limited their willingness to engage in the toolkit package. Time limitations were perceived as particularly problematic for clinicians and impacted their intention to introduce the toolkit to clients. Furthermore, some clinicians worried about the perceptions of clients in relation to time:

*So you know one of the clients that we had was, “Well you know, is it going to take away from my therapy time? Like, am I going to have to sit down and do it with you?”* (Clinician)

However, other clinicians felt that the client toolkit legitimised *spending time* to discuss what was important with clients.

A few clients with acute health events had many other demands on their time and attention, and as such the introduction of the client toolkit during their hospital stay was perceived as, *“too much information; too much overload”* (Client). This view was echoed by some clinicians from the same setting. Another concern was that certain questions were *“tricky”* because *“It’s a big question to ask.”* (Client). Clinicians suggested that adding examples of possible responses to the questions could provide some direction to clients.

Clinicians’ engagement with the toolkit package was influenced by its resonance with their philosophy of practice. In particular, they liked the way in which the client toolkit focused on the person rather than the condition:

*I think this is really good in providing that ability for them to kind of express what’s important to them and what they, to focus more on just themselves and who they are.* (Clinician)

The ADAPT acronym produced as a prompt card was universally liked by clinicians:

*I still have mine on my desk because I think that’s such an awesome prompt is, ‘What is it I need? What do they want from me?’ I think that’s awesome […] and that’s just client centred practice*. (Clinician)

In some instances, clients had already developed their own strategies to identify and communicate what was important to them. In these cases, while the philosophy of the client toolkit was consistent with their own way of operating and thinking, it did not necessarily provide additional value for them:

*I’ve reassessed what is important now in my life and yes one does do that. So it’s beneficial for me from that point of view but I’ve done [that] already without utilising the toolkit.* (Client)

For others, the client toolkit presented a useful prompt, to help them more explicitly think about aspects of their healthcare that were important to them:

*…yes in hindsight yes. Things I’ve re-tackled, I’ve reassessed what is important now in my life…* (Client)

Only a small number of clients actually translated these thoughts into written form in the client toolkit. Several clients raised the importance of trust in sharing information and their concern about the privacy of the client toolkit was a barrier to committing their reflections to paper.

Although clinicians and clients generally found the toolkit package to be consistent with their thinking and approach, both groups also identified a number of barriers that limited investment of time, energy and work to an extent where active uptake was modest.

### Collective action

Collective action refers to the work that users have to do to use the toolkit package effectively. It includes questions seeking to understand how compatible the toolkit package is with existing work practices, how much training is needed to use it and whether it will enhance or impede clinical work.

The majority of clinicians felt the ADAPT acronym served as a useful reminder of a person-centred interaction style and felt it enhanced their work. Furthermore, all clinicians expressed a familiarity with the content and perceived intent of the toolkit package and as such, the toolkit package was perceived to have a good fit with their clinical practice. Some clinicians recognised the client toolkit to be a structure to support a person-centred approach and believed it had the potential to improve related processes.

*But for me it definitely, like that goal setting was definitely moved forward I guess in terms of my priorities about what to talk to my client about. Making sure that what I was doing was relevant to them.* (Clinician)

One client expressed doubt that the toolkit package would be sufficient to make a difference to clinicians who are *"just going through motions*" or who *"don’t listen to you"*. Clients did not offer any views on whether they perceived use of the client toolkit as extra effort.

The electronic layered clinicians’ resource, which expanded on the ADAPT principles with more detail and clinical examples, however, was barely used with limited time and the type of format (necessitating deliberate time at a computer) reported as the main barriers for use.

Although clinicians initially seemed to grasp the information provided about the toolkit package in the brief training session, on reflection, many perceived the information to be too theoretical and abstract for them to easily incorporate into clinical practice and described having to work out how to introduce, describe and facilitate use of the toolkit. One clinician (also an implementation champion) suggested that more time and explanation from the research team would help clinicians to better understand the purpose of the client toolkit and how they could best support clients in its use. Another clinician voiced an opinion that the toolkit package would be easier to use if it could be better integrated into existing processes. On the other hand, there were a few clinicians who described the toolkit package as having a natural fit with their practice. Disciplines such as occupational therapy and clinical psychology seemed to find it easier than physiotherapists to integrate the toolkit package into clinical practice and support the client in their use of the client toolkit.

In summary, clinicians felt the ADAPT acronym enhanced their practice, but that they had insufficient knowledge and skills to adequately introduce and support clients in their use of the client toolkit. Furthermore, where use of the toolkit package required work over and above existing practices (e.g. perceived as an additional task to do, needing extra or dedicated time and access to a computer to explore the electronic resource) clinicians were less inclined to use it.

### Reflexive monitoring

Reflexive monitoring refers to the opportunities users have to appraise the benefits and costs of the intervention. This includes whether the toolkit package has had any effect, whether it can be adapted if needed and how users view the toolkit package when it has been in use for a while.

Within the timeframe of this pilot study, there was limited opportunity for appraisal of the effects of the toolkit package or to capture views of its long-term use. However, our intention from the outset of the entire project was for the views and reflections of users to shape the design and use of the toolkit package, but the refinements were not obvious to the participants of this phase, as they occurred subsequent to participants’ feedback.

We received specific suggestions from both clients and clinicians related to the medium of the client toolkit; an electronic version was preferred by many, though for different reasons. As noted already above, privacy concerns related to writing on paper were raised by one client. Another client referenced their ability to write: *“something electronic would be my preference, to type what I’m* saying” (client). Clinicians expressed their desire for an electronic version to more easily meet their clinical record keeping obligations.

Users were generally positive about the design and size of the client toolkit, but we received mostly negative feedback about the ring used to bind the pages of the client toolkit together; most people commented on how difficult it was to open and close in order to add more sheets (Fig. 1).

In summary, the research process engaged in this study explicitly sought feedback from participants to support refinement of the toolkit package. Although users adapted the client toolkit to their unique needs and situations, the refinements to the toolkit package based on the collective feedback occurred after participants’ involvement and so were not evident to participants of this phase.

### Refinement of the toolkit package

Our findings offer useful insight into aspects of the toolkit package relevant to future uptake from the perspective of clients and clinicians, including perceived value, its operational value and consideration of the client toolkit’s form and function. NPT provided a useful framework for analysis in that it allowed us to carefully consider the findings and then respond with respect to coherence, cognitive participation, collective action and reflexive monitoring [20]. These constructs provided direction for the refinements of the toolkit package which are outlined below.

We developed two short introductory videos to simplify the introduction of the toolkit package and to illustrate alternate ways the client toolkit could be used, in keeping with the intended flexibility of the client toolkit and also to satisfy the need for tangible examples. One video is specifically for clinicians [23] and the other video speaks to potential users of the client toolkit and their families [24]. We reasoned clinicians could also use the client video when introducing the client toolkit to clients. Both videos are freely available and can be watched multiple times if needed. Our hope was that these changes would improve the sense of coherence for both clients and clinicians by clarifying the purpose and offering a range of examples of ways in which the client toolkit might be used.

**Fig. 3:**
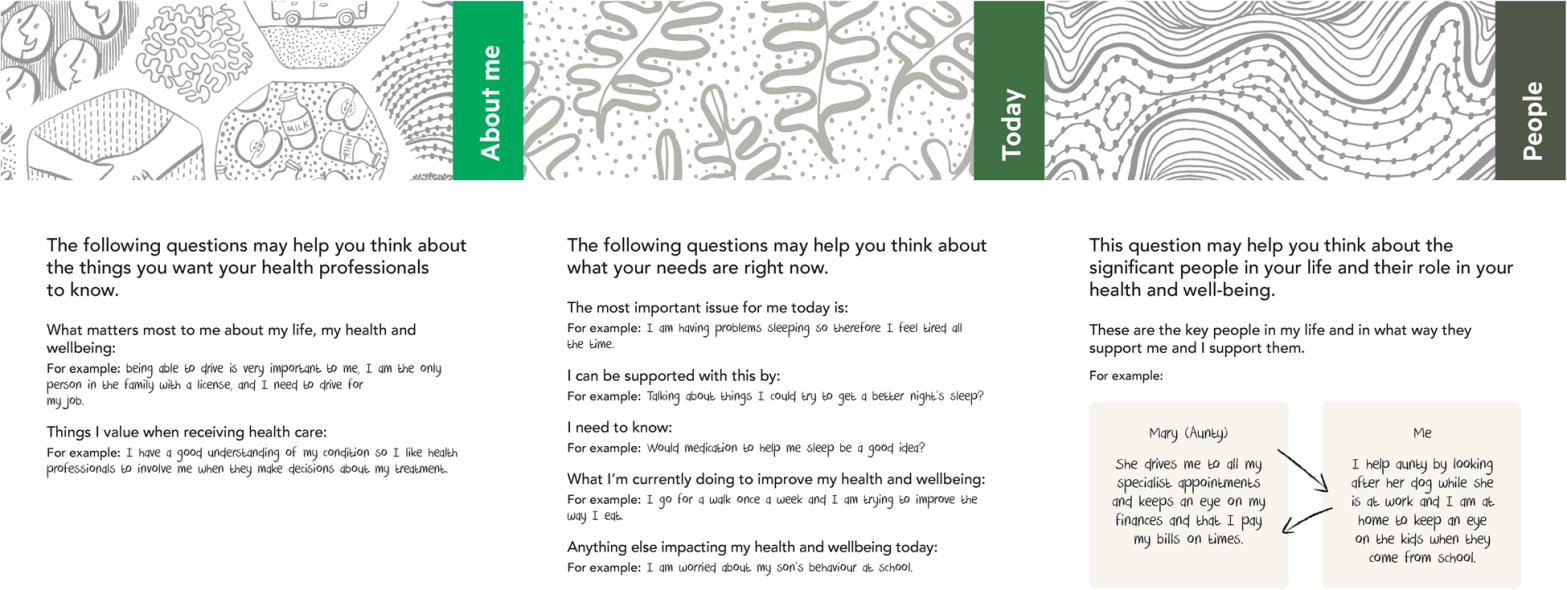
Client toolkit (prompt version). Developed by our team for this study

We also made refinements to the client toolkit; we simplified the language and condensed several questions that were not able to be easily distinguished and to minimise the number of “*big questions*”. We developed examples of responses to all the questions to illustrate potential responses (Fig. 3). Given the client toolkit was commonly used by clients as a prompt, rather than as a written record of client needs and preferences, we developed an alternative short form ‘prompt’ version of the toolkit with the same questions but without space to record the responses (Fig. 3). This fold out version was smaller to handle and was not bound together with the ring which many clients had found problematic. We aimed to increase the engagement of clients and clinicians with these changes intending to minimise the identified barriers to cognitive participation.

To address the need for more comprehensive training identified by clinicians, we developed a reflective training package, presented in three modules. The modules were intended to be interactive and relied on discussion and reflection from participants. Each module was a pre-requisite for subsequent modules. The first ‘bronze’ module was intended for all staff, including non-clinical staff. Our reason for including all staff was to make explicit that a whole of service approach is necessary to ensure the principles and intent of the toolkit package can be embedded. As such, buy-in from all staff was important. The aim of the ‘bronze’ module was to relate the staff’s own experience to the central orienting question of toolkit package: ‘Who is this person and what do they need from me today?’ The second ‘silver’ and third ‘gold’ modules were intended for clinicians who would be responsible for introducing the client toolkit to clients. The ‘silver’ module aimed to build an understanding of the practical application of the toolkit package. It included two purpose-built videos of clinical scenarios to specifically demonstrate the use of the ADAPT style in practice. The two videos were deliberately designed to present two contrasting scenarios – one reflecting a more conventional approach to practice and the other presenting an approach consistent with the ADAPT principles. Following the ‘silver’ module clinicians were encouraged to introduce and support the client toolkit in practice. The ‘gold’ module (carried out at least two weeks later to allow clinicians time to cumulate some experiences of use of the toolkit package) then focused on providing an opportunity to reflect on their experiences and draw on their shared experiences to develop strategies to support sustained use of the toolkit package in practice moving forward. Clinicians were encouraged to share both successes and challenges they had experienced in their use of the toolkit package. We additionally showed a further two videos we had produced to present different clinical scenarios to form the basis for identifying specific challenges encountered and discuss solutions for supporting use of the client toolkit. The development of the training modules was aimed to improve greater coherence for clinicians and more collective action which included explicit discussion and rehearsal of fit with own style and practice.

## Discussion

Generally, the philosophy of the toolkit package resonated with the values and beliefs of participants and this alignment was crucial for both clients and clinicians before committing further. In some instances, clinicians recognised the added value of the structural support provided by the client toolkit to enhance aspects of their work such as goal setting, which even led some clinicians to critically reflect on how they had previously worked. However the same agreement with the underlying principles led to a different response from other clinicians, who, similar to responses described in other studies, felt they already worked in a person-centred way [25], leading to their dismissal of the toolkit package as superfluous [26], which was similar to the views of some clients. Clinicians with such views did not recognise any scope for improvement to their clinical practice, obviating any need for the toolkit package rather than stating the toolkit package did not meet their need in this area. Such a view implies that person centred practice can be achieved, in contrast with a perspective that considers person-centredness less as a dichotomy and more as a continuum. Person-centredness as a continuum is a position that allows, even encourages, the understanding that continual improvements are always possible [13, 27].

We assumed that clinicians who had volunteered to participate in this study were likely to be predisposed to person-centred practice. Consequently we were surprised to see these two divergent responses to the toolkit package. It may be that these responses highlight differing degrees of critical reflection [13, 28–30]. Alternatively, the concept of collective action highlights the work that clinicians have to do to integrate interventions into practice [20], which may have been limited. Banja and Eisen discuss reasons clinicians may not be prepared to do such work; including the quality of rehabilitation research, being resistant for personal reasons to an intervention and organisational barriers to implementation [31]. One or more of these reasons may have contributed to clinicians dismissing the toolkit package in the current study.

In general, it appears that if clinicians perceived that a component of the toolkit package was easily integrated into practice or augmented existing processes (e.g. goal setting) and they perceived an immediate benefit (to themselves or the client) then they were more likely to continue to use it, similar to the findings of Kennedy et al. [26]. On the other hand, use of the client toolkit was not sustained when clinicians perceived it detracted from ‘doing’ therapy, which may reflect a difference in how clinicians viewed the value of ‘talking’ rather than ‘doing’ [12, 32].

The intended flexibility regarding who, how and to what extent the client toolkit can be used made it difficult for clinicians and clients to be clear about its purpose. This lack of coherence limited uptake in both groups in this pilot study. We aimed to improve clarity by designing introductory videos that included a simple explanation and examples illustrating the flexible use; one video was specifically for clients and families and the other for clinicians. However, we acknowledge that this response may ultimately be inadequate and the flexibility, although clearly an ideal and desirable feature to fit a wide range of situations [14], may be difficult to balance with the sense of being individualised enough for any one unique user or purpose [5]. The flexibility in application may also continue to be challenging for clinicians, who, in this phase, were responsible for introducing and supporting use of the client toolkit; a finding not dissimilar to other studies [30]. The collective action required to operationalise an intervention into practice may be simpler when it is tailored more specifically for a narrowly defined purpose or group of users. It is conceivable that there will always be some tension between the flexibility of intended purpose and the extent to which the toolkit package can meet specific needs. This balance must be carefully considered and negotiated when developing a tool.

Clinicians were explicit in the first phase of this project that the uptake of any intervention was dependent on time involved [14] and this was the reasoning for producing the principles of the toolkit package as an abbreviated prompt card. Indeed, the prompt card was the most liked and used part of the clinicians’ resource. In contrast, the extended clinicians’ resource (interactive pdf) was barely used with limited time and format (necessitating deliberate time at a computer) reported as the main reasons it was not used. Time is universally named as a barrier in any change or service improvement initiative [33–36], however NPT provides further insight into how time acts as a barrier linking it with cognitive participation. ‘Not enough time’ can be understood as the shorthand clinicians use when they are not prepared to invest time into understanding and/or operationalising a new practice. If clinicians’ cognitive participation is limited, they will not be able to use the intervention effectively, which will further contribute to a negative assessment of value (collective action). Simply allocating time, without assessing first whether and how an intervention has a philosophical fit with values of clinicians (or a service), is unlikely to be a successful strategy.

The refinements to the toolkit package described above in conjunction with the development of a training package for clinicians are currently being trialled in the next phase of the project and we anticipate that they will go some way towards addressing issues of implementation. We plan to address ease of integration by working closely with the clinical services taking up the toolkit package to look at their specific service structure and explore further refinements specific to their service, including integration with their usual practices, workflows and processes. Although we had buy-in from the services who supported the project from conception and participated in the previous phase, we recognise that uptake is likely to be dependent on our continued responsiveness to the services’ needs [17] and subsequent adaptation of the toolkit package [37]. We also acknowledge however that implementation is a complex process with many other factors that influence the uptake of an intervention, and which are potentially outside the scope of our influence in this project [31, 37].

We appreciate that this study has limitations. An implementation project is by necessity responsive; so while the methods and results are portrayed in a linear manner here, some of our refinements to the toolkit package were implemented towards the end of the pilot phase. Rather than discuss the acceptability of the individual refinements in this paper, we plan to report on the acceptability of the toolkit package in its entirety, in a subsequent paper on the results of the larger implementation phase.

There are also some limitations in our sample; our patient participants lived with four different neurological conditions, all of which involved an acute onset. As such, our findings may not resonate with people with other neurological conditions, particularly those with a gradual onset and/or a progressive course. We also attempted to gain breadth with respect to disciplinary background of clinicians, however given our small sample, only one occupational therapist and one clinical psychologist participated. As such, we have been careful to avoid drawing strong conclusions about particular disciplinary perspectives. We also recognise that our recruitment strategy and findings may have been influenced by the power relationships inherent in a healthcare hierarchy (senior clinician recruiting clinicians and clinicians recruiting clients), potentially overinflating uptake, particularly amongst clinicians. It is also possible that these power relationships may have impacted the perspectives offered. Therefore, the findings should be interpreted with this potential in mind.

## Conclusions

There was widespread support for the principles underpinning the toolkit package from clients and clinicians. However, it was less clear how the client toolkit could support these principles in clinical practice impacting buy-in. The intended flexibility of use of the client toolkit that we encouraged made it difficult for clinicians and clients to be clear about its purpose and for clinicians to operationalise in practice. Clinicians and clients identified a number of barriers that limited the time, energy and work users were able or prepared to invest, to the extent that uptake of the toolkit package was modest. Use of the toolkit package appeared more likely when clinicians perceived it to augment existing processes (e.g. goal setting) rather than detract from ‘doing’ therapy. Understanding how supporting the client toolkit could add value to the therapeutic encounter was necessary for clinicians to invest time and perceive the worth of the toolkit package.

NPT provided a useful analysis framework to consider and respond to the findings with respect to coherence, cognitive participation, collective action and reflexive monitoring. Refinements to the toolkit package included addition of introductory videos to improve the understanding of the client toolkit and its inherent flexibility. We simplified the language, condensed questions and produced an alternate ‘prompt’ version of the client toolkit to minimise the barriers for investment of time, energy and work in its use. We also developed a modular training package for clinicians to improve coherence and the sense of work required to use the toolkit package. We hope the refinements we have made to the toolkit package will improve uptake for both clinicians and clients.

## Supplementary information

**Additional file 1.** Client interview guide.

**Additional file 2.** Clinicians’ Focus Group/Interview Guide.

## Data Availability

The datasets generated and/or analysed during the current study are not publicly available due to information contained therein that could compromise research participant privacy but are available from the corresponding author (SM) on reasonable request.

## Abbreviations

ADAPT: Assume nothing, Discuss, Acknowledge expertise, Promote partnering, Tailor Care
NPT: Normalisation process theory
