# Supplementary material for: Pilot trial of The Living Well Toolkit: qualitative analysis and implications for refinement and future implementation
Source: BMC Health Serv Res. 2020 Jan 30;20:69. doi: 10.1186/s12913-020-4920-5 (PMC6993322; doi:10.1186/s12913-020-4920-5)
Supplement: Supplementary file 1 — Additional file 1. Client interview guide. [file 12913_2020_4920_MOESM1_ESM.docx]

Client interview guide

**Start-up conversation:** It has been a week since you were first introduced to the toolkit – what are your initial thoughts on the toolkit?

**Example questions:**

a) Have you used the toolkit?

b) What worked for you or did you find helpful?

c) How have you used the toolkit so far (have they used it for themselves to have they used it with someone)?

d) Who did you use the toolkit with?

e) If you have used it, has it changed anything in the way you interacted with your health professional? Or Did the toolkit help you to share information with those?

f) How did you feel about using the toolkit?

g) If you haven’t used it, why do you think that might be –what stopped you from using it? OR what did you not like about it (if person indicates that they don’t like the toolkit).

h) How do you think you will use the toolkit after your discharge from this setting?

i) What do you think about the design?

**Prompts for type of information we are looking for:** how was the toolkit introduced, what section was most useful to the person and why, did they understand the purpose of the toolkit, what are the participants views on the format, design, usability, size font etc.
