# Supplementary material for: Pilot trial of The Living Well Toolkit: qualitative analysis and implications for refinement and future implementation
Source: BMC Health Serv Res. 2020 Jan 30;20:69. doi: 10.1186/s12913-020-4920-5 (PMC6993322; doi:10.1186/s12913-020-4920-5)
Supplement: Supplementary file 2 — Additional file 2. Clinicians’ Focus Group/Interview Guide. [file 12913_2020_4920_MOESM2_ESM.docx]

Clinicians’ Focus Group/Interview Guide

1. Training requirements:
   1. In view of wider implementation and informing health professionals about the toolkit and its purpose, what’s your experience of the information you received?
   2. Were the instructions clear?
   3. Was any further information on the toolkit, its purpose or use needed?
2. Experience with recruitment and toolkit introduction:
   1. Can you tell us about your experience of introducing the toolkit to your client?
   2. How easy was it for the client to understand its purpose and did they require much support with its use?
   3. What do you consider as the key barriers to implementation of the toolkit?
   4. Were there any major flaws in the toolkit that question its usefulness or that would stop its wider implementation?
3. Experience with client toolkit:
   1. Was what the client toolkit influential in the way you interacted with the client?
   2. How did the toolkit inform your practice?
   3. What were the benefits or drawbacks in using the toolkit in your practice?
   4. Can you recommend two things that would make the toolkit more useful?
   5. What is your opinion on the format and the content of the toolkit?
4. Experience with clinician’s resource:
   1. What is your feedback on the clinician’s resource?
   2. How useful did you find the PDF and the diary sized reminders?
   3. How did it inform your interaction with the clients?
5. Recommendations for wider implementation:
   1. What did you like about the toolkits in general?
   2. What did you not like?
   3. How would you improve it?
